# Autonomy support, basic needs satisfaction, and involvement in physical education among Norwegian secondary school students

**DOI:** 10.3389/fpsyg.2024.1505710

**Published:** 2024-12-10

**Authors:** Amund Langøy, Åge Diseth, Bente Wold, Ellen Haug

**Affiliations:** ^1^Department of Teacher Education, NLA University College, Bergen, Norway; ^2^Department of Health Promotion and Development, University of Bergen, Bergen, Norway; ^3^Department of Education, University of Bergen, Bergen, Norway

**Keywords:** basic psychological needs in PE scale, participation in physical education, gender differences, physical activity, PE teacher

## Abstract

**Purpose:**

This study investigated the relationship between teacher autonomy support, students’ basic psychological needs satisfaction, and involvement in physical education (PE) with gender specific analyses. Additionally, the study examined the validity of a Norwegian version of the Basic Psychological Needs in PE (BPN-PE) scale.

**Method:**

Survey data from the Norwegian 2017/2018 Health Behaviour in School-aged Children study.

**Results:**

A structural equation model showed that perceived autonomy support from teacher predicted students’ basic needs satisfaction of competence, autonomy and relatedness. Furthermore, satisfaction of competence predicted weekly PE participation and physical activity during PE among boys. The analyses revealed mean level differences with boys scoring higher than girls on all the investigated variables. The findings also supported the validity and reliability of the BPN-PE scale across genders.

**Discussion/conclusion:**

The study adds knowledge to the understanding of the relationship between autonomy support from teachers, students basic need satisfaction and students’ involvement in PE.

## Introduction

School physical education (PE) has been identified as an important arena for physical activity (PA) (Sallis et al., 2012; World Health Organization, 2017; Ryu et al., 2020), that can contribute to the overall levels of PA recommended to enhance health (Chaput et al., 2020). PE may contribute to the development of physical competence, fundamental movement skills, social skills, self-esteem, and pro-school attitudes (Bailey, 2006). Participation in PE (Shephard and Trudeau, 2000) and attitudes to PE (Kjønniksen et al., 2009) have also been associated with more favorable PA patterns (OECD, 2019; Tassitano et al., 2010; Uddin et al., 2020) and with PA later in life (Kirk, 2005; Trudeau and Shephard, 2005). However, the literature suggests that not all students participate in PE (Sälzer and Heine, 2016) and that some skip classes without a valid reason (Moen et al., 2018). Also, studies show that moderate to vigorous physical activity (MVPA) declines with age during adolescent years in PE lessons (Burns et al., 2015; Cheval et al., 2016), and in overall PA (World Health Organization, 2020).

There is a considerable variation in perceptions of and motivation for PE among secondary school students (Gråstén and Watt, 2017; Huhtiniemi et al., 2019; Säfvenbom et al., 2015; Vasconcellos et al., 2020). Students have different experiences of support for and satisfaction of psychological needs for competence, autonomy and relatedness. According to self-determination theory (SDT), these needs are important for motivation and wellbeing (Ryan and Deci, 2017). Previous research findings have shown that they promote different PE outcomes (Vasconcellos et al., 2020). Thus, the study of BPN satisfaction in PE is considered central to our understanding of adolescents’ engagement in the subject (Erdvik et al., 2020). However, most studies have assessed the relationship between BPN satisfaction and different psychological constructs [e.g., global self-worth, self-concept and vitality (Erdvik et al., 2019; Garn et al., 2012; Taylor and Lonsdale, 2010)] and only a few have examined how BPN satisfaction is related to measures of PE participation (Taylor and Lonsdale, 2010; Taylor et al., 2010). To add more knowledge on this direct relationship the main focus of this study is to examine how Norwegian 10th-grade students’ perceived autonomy support (from the PE teacher) is associated with BPN in the PE setting, and how students’ BPN satisfaction is related to PE involvement. Furthermore, it is of scientific and practical interest to evaluate the psychometric properties of an increasingly applied measure of BPN in PE for use in a different cultural context. Therefore, the Basic Psychological Needs in PE (BPN-PE) scale will be validated among Norwegian 10th-grade students as a part of this study.

Self-determination theory assumes that the psychological needs for autonomy, competence and relatedness must be satisfied to experience subjective wellbeing, psychological growth, and integrity (Deci and Ryan, 2000; Hagger et al., 2003; Ryan and Deci, 2017). To experience satisfaction of the need for competence in the PE context, students must experience understanding for and mastery of the tasks presented to them. They also need to feel that they can develop their skills during PE classes (Standage et al., 2005). Students’ need for autonomy is satisfied when they experience ownership of their actions for self-organized experiences, while satisfaction of relatedness requires that students experience a sense of belongingness to their classmates, which means that they need to feel connected and involved with others (Ryan and Deci, 2017).

Gender differences have been indicated in students’ needs satisfaction in PE (Vasconcellos et al., 2020). Some studies have reported higher levels of need satisfaction in PE for boys than girls regarding autonomy needs (Hosseini et al., 2020; Mouratidis et al., 2015) and competence needs (Bagøien et al., 2010; Hosseini et al., 2020; Mouratidis et al., 2015). Regarding gender differences in satisfaction of relatedness needs in PE, previous research has shown mixed findings. Some studies have found that girls report higher levels of relatedness need satisfaction (Bagøien et al., 2010; Gråstén and Watt, 2017), other studies have shown lower levels among girls (Ntoumanis and Standage, 2009; Ullrich-French and Cox, 2014), and some studies have shown no gender differences in relatedness satisfaction among school students (Mouratidis et al., 2015; Xiang et al., 2017).

According to the self-determination theory, autonomy support facilitates satisfaction of BPN (Ryan and Deci, 2017; Vallerand et al., 1997). An autonomy-supportive teacher uses a noncontrolling language, provides the students with choices and options, tries to understand how students cope with tasks, and provides supplementary help (Deci and Ryan, 1985; Hagger and Chatzisarantis, 2007). While some studies have shown that boys report higher perceived autonomy support than girls (Bagøien et al., 2010; Hosseini et al., 2020; Mouratidis et al., 2015;), others have shown no gender differences (Shen et al., 2008).

Several studies have found that autonomy support from teachers positively predicts satisfaction of basic psychological needs during PE (Bagøien et al., 2010; Garn et al., 2012; Haerens et al., 2015; Ntoumanis, 2005; Standage et al., 2005; Taylor and Lonsdale, 2010; Wang, 2017). Some studies have also shown positive associations between autonomy support from PE teachers, satisfaction of BPN in PE and favorable PE outcomes such as subjective vitality (Taylor and Lonsdale, 2010), concentration (Erturan-İlker et al., 2018), physical self-concept (Garn et al., 2012). Regarding involvement in PE, BPN satisfaction have been found to be positively associated with students’ effort during PE (Taylor and Lonsdale, 2010; Taylor et al., 2010). Gråstén et al. (2021) also found a direct positive association between satisfaction of the need for relatedness and moderate to vigorous physical activity (MVPA) in PE among both boys and girls, and an indirect positive association between satisfaction of the need for competence and MVPA via extrinsic motivation. Studies have found gender differences in PE participation with girls participating less than boys (Martins et al., 2020). Girls also experience more barriers in PE which can discourage their participation in PE (O'donovan and Kirk, 2008; Røset et al., 2020; White et al., 2021).

This study will add to the existing literature by investigating the direct relationship between BPN satisfaction on both weekly PE participation and physical activity during PE, with gender-specific analysis. Also, the study will validate the Norwegian version of the Basic Psychological Needs in PE (BPN-PE) scale, developed by Vlachopoulos et al. (2011) to meet the need for a psychometrically sound measure of basic needs satisfaction in PE. Positive associations between perceived teacher autonomy support and students’ basic needs satisfaction and involvement in PE will add support to the nomological validity of the BPN-PE scale (Hagger et al., 2017). It is of scientific and practical interest to evaluate its psychometric characteristics and variance across diverse populations (Sánchez-Oliva et al., 2018). So far, the scale has been validated in several countries (Cagas and Chasandra, 2014; Huhtiniemi et al., 2019; Menéndez Santurio and Fernández-Río, 2018; Sánchez-Oliva et al., 2018), but not yet in any Scandinavian country, and measurement invariance across genders has been assessed only to a limited extent (Sánchez-Oliva et al., 2018; Vlachopoulos et al., 2011).

Thus, based on theoretical assumptions and previous research findings, the following hypotheses were proposed:

1. Autonomy support from teacher is positively associated with students’ basic needs satisfaction in PE, which in turn is positively associated with weekly PE participation and physical activity during PE.
2. There are gender differences in students perceived autonomy support and in their satisfaction of the needs of autonomy, competence and relatedness in PE.
3. The Norwegian BPN-PE scale is a valid and reliable instrument across genders.

## Methods

### Sample

The sample used in the study stems from the Norwegian 2017/2018 survey of the Health Behaviour in School-aged Children (HBSC) study, a World Health Organization collaborative national study (Inchley et al., 2020). The participants comprised 882 students (10th grade, 15–16 years old, 423 boys, 71% response rate) who responded to a survey. The primary sampling unit was school classes (*n* = 67), and the schools were chosen from a geographically stratified list, to ensure a nationally representative sample. Students in classes with fewer than four students were removed (*n* = 24), reducing the total number of participants to 858. Participation was voluntarily, and the students responded anonymously. The class teachers administered the survey during a school lesson.

### Measures

#### Autonomy support

A Norwegian version of the Sport Climate Questionnaire was used to measure students’ perceived autonomy support from teachers. This version was translated by Ommundsen and Kvalø (2007) from the 15-item Sport Climate Questionnaire. They modified the wording slightly to fit the PE context. In this survey, the short six-item version of the scale was applied. Hagger et al. (2003) found internal reliability (Cronbach’s alpha) for this version to be 0.93. A sample item is “I feel that the PE teacher provides us with choices and options.” Participants responded to the items using a Likert-type scale ranging from 1 (Strongly disagree) to 7 (Strongly agree). Cronbach’s alpha for autonomy support for the present study was 0.91, which is acceptable and consistent with other studies (Hagger et al., 2003; Ommundsen and Kvalø, 2007).

#### Basic needs satisfaction in physical education

A Norwegian version of the BPN-PE scale was used (Vlachopoulos et al., 2011). This 12-item scale was originally developed and tested in a Greek context in a sample of 10–18-year-olds (Vlachopoulos et al., 2011). Sample items are “We do things that are of interest to me” (Autonomy), “I feel that I improve even in the tasks considered difficult by most of the children” (Competence), and “My relationships with my classmates are very friendly” (Relatedness). The response scale was a seven-point Likert scale ranging from 1 (I do not agree at all) to 7 (I completely agree) with the midpoint of 4 using the verbal anchor (I moderately agree). The scale was translated according to standardized translation procedures adapted from the HBSC study. The scale was translated into Norwegian and then backtranslated to English according to the standard translation approach within the HBSC study, with direct translation and adaptations permitted only when absolutely necessary for linguistic clarity (Inchley et al., 2018). Beyond direct translation, the HBSC procedures emphasize the need to adapt questions to fit cultural contexts such as modifying examples or terminology. This was, however, not considered relevant for the translation of the BPN-PE scale into Norwegian. Previous research has supported a three-factor structure (Cagas and Chasandra, 2014; Menéndez Santurio and Fernández-Río, 2018; Pires et al., 2010; Sánchez-Oliva et al., 2018; Vlachopoulos et al., 2011), internal consistencies for BPN-PE (Cagas and Chasandra, 2014; Vlachopoulos et al., 2011), and acceptable reliability (Huhtiniemi et al., 2019; Pires et al., 2010; Sánchez-Oliva et al., 2018). Research has also shown adequate inter-item correlation and acceptable item-total correlation (Cagas and Chasandra, 2014; Menéndez Santurio and Fernández-Río, 2018; Pires et al., 2010).

#### Weekly physical education (PE) participation

In Norway, 10th-grade students would normally have two sessions of 45 min of mandatory PE per week but can also chose elective classes. To measure how often the students participated in PE, we included the following item: “How many times in a normal week do you participate in physical education? (Also incorporate elective subjects such as sports and outdoor life). Tap one 90 min class as two times.” The response scale was labeled 1 (0 times), 2 (1 time), 3 (2 times), 4 (3 times), 5 (4 times), and 6 (more than four times). Previous research has found this item to be positively associated with autonomous motivation (Haug et al., 2023).

#### Physical Activity (PA) during physical education (PE)

To measure physical activity during physical education, we incorporated a physical activity screening measure for use with adolescents (Prochaska et al., 2001). Previous research has shown that this measure is positively associated with autonomous motivation in PE (Haug et al., 2023). The wording of the question was: “How many minutes in a single PE class (45 min) do you usually perform physical activity in a way that makes you warm and out of breath?.” The response scale was labeled 1 (0 min), 2 (1–10 min), 3 (11–20 min), 4 (21–30 min), and 5 (more than 30 min).

### Ethics approval and consent to participate

The HBSC study was approved by the Norwegian Centre for Research Data. Active consent was sought from both children and their parents. A detailed information letter was given both in paper form and electronically to parents or custodians for all participants. Consent to participate was given by e-mail, SMS, or signing and returning the form to the teacher.

### Statistical analyses

Exploratory statistical analyses were performed using IBM SPSS 26.0 (2019). Confirmatory factor analysis (CFA) and structural equation modeling (SEM) were performed using IBM SPSS AMOS 25.0 (2018) to test the structural relationship between the latent variables described in the Introduction. The fit indices chi-square/degrees of freedom (df) ratio, comparative fit index (CFI), and the root mean square error of approximation (RMSEA) were determined to investigate the appropriateness of the CFA and SEM analyses (Muthén and Muthén, 2012). The CFI should ideally be close to 0.95, but above 0.90 may be acceptable. The RMSEA should ideally be below 0.05, but below 0.08 is acceptable. The chi-square/df ratio should ideally be <2 (Byrne, 2010). However, chi-square statistics tend to underestimate model fit in larger samples, such as this one (Schumacker and Lomax, 2004), and this index will therefore not be considered critical in the analyses.

## Results

### Measurement model for the BPN-PE scale

To produce a measurement model for the BPN-PE scale, CFA was conducted, in which the three latent factors of autonomy, competence, and relatedness accounted for the particular items in this scale. Factors were allowed to correlate, but no correlated residuals were permitted. The CFA produced acceptable fit indices (*χ*^2^ = 481.58, df = 51, *p* < 0.001, *χ*^2^/df = 9.44, CFI = 0.95, RMSEA = 0.10). To investigate configural invariance between genders, a multigroup CFA was performed. This analysis showed acceptable fit indices (*χ*^2^ = 550.99, df = 102, *χ*^2^/df = 5.04, CFI = 0.95, RMSEA = 0.07), thus supporting configural invariance between boys and girls for this measurement model. To investigate metric invariance, the model was constrained to be equal across genders (*χ*^2^ = 574.81, df = 114, *χ*^2^/df = 5.04, CFI = 0.95, RMSEA = 0.03). A chi-square difference test showed that the difference between the unconstrained and the constrained model was not significant at the 1% level, but the difference was significant at the 5% level (*χ*^2^ diff = 23.82, df diff = 12, *p* = 0.02). However, there was no difference in CFI values between the unconstrained and the constrained model. Hence, these analyses provided sufficient support for configural and metric invariance.

### Measurement model for the autonomy support scale

A measurement model of the autonomy support scale was produced using CFA. In this analysis, error-covariance were imposed between the two items “I feel that my PE teacher provides us with choices and options” and “I feel understood by my PE teacher” (items 1 and 2) as well as the two items “My PE teacher listens to how I would like to do things” and “My PE teacher tries to understand how I see things before suggesting a new way to do things” (items 5 and 6) because of the content similarities between these items. This model produced acceptable fit indices (*χ*^2^ = 53.63, df = 7, *p* < 0.00, *χ*^2^/df = 7.66, CFI = 0.99, RMSEA = 0.09). To explore configural invariance across genders, a multigroup CFA was performed. This analysis showed acceptable fit indices (*χ*^2^ = 64.57, df = 14, *χ*^2^/df = 4.6, CFI = 0.99, RMSEA = 0.07).

To investigate metric invariance, the model was constrained to be equal across genders (*χ*^2^ = 74.47, df = 20, *χ*^2^/df = 3.72, CFI = 0.99, RMSEA = 0.06). A chi-square difference test showed that the difference between the unconstrained and the constrained model was not significant (*χ*^2^ diff = 9.90, df diff = 6, *p* = 0.13). Taken together, these results supported configural and metric invariance for the autonomy support scale.

### Descriptive statistics and correlations

Descriptive statistics and correlations between all variables for the total sample and boys and girls separately are presented in Table 1. Reliability coefficients (Cronbach’s alpha) are shown diagonally. The correlation analysis showed that teachers’ autonomy support correlated significantly with the satisfaction of all three needs and moderately with physical activity (PA) during PE, but no correlation was observed with weekly PE participation all three needs were moderately correlated with PA during PE and weekly PE participation. Skewness and kurtosis for all variables were within the recommended values between −2 and +2 (George and Mallery, 2010), which supports normal univariate distribution. Mean-level gender differences were analyzed using independent sample t-tests. The results (Table 1) show that boys scored significantly higher than girls on all variables.

**Table 1:** Descriptive statistics and correlations between variables.

	*M*	SD	Observed range	Skewness	Kurtosis	1.	2.	3.	4.	5.
1. Autonomy support	3.32	1.08	1.0–5.0	−0.30	−0.62	*0.938*				
Boys	3.45	1.05	1.0–5.0	−0.41	−0.39					
Girls	3.21	1.10	1.0–5.0	−0.20	−0.74					
t-test	3.00**									
2. Competence	4.51	1.52	1.0–7.0	−0.32	−0.31	**0.563**	*0.943*			
Boys	4.83	1.43	1.0–7.0	−0.44	−0.09	**0.530**				
Girls	4.20	1.53	1.0–7.0	−0.20	−0.50	**0.574**				
t-test	3.57**									
3. Relatedness	4.89	1.49	1.0–7.0	−0.64	0.00	**0.502**	**0.757**	*0.913*		
Boys	5.10	1.43	1.0–7.0	−0.72	−0.33	**0.489**	**0.773**			
Girls	4.70	1.52	1.0–7.0	−0.58	−0.21	**0.496**	**0.730**			
t-test	3.62**									
4. Autonomy	4.26	1.51	1.0–7.0	−0.26	−0.44	**0.668**	**0.822**	**0.755**	*0.916*	
Boys	4.56	1.48	1.0–7.0	−0.39	−0.14	**0.674**	**0.805**	**0.777**		
Girls	4.00	1.50	1.0–7.0	−0.18	−0.61	**0.648**	**0.825**	**0.718**		
t-test	5.01**									
5. Weekly PE part.	3.62	1.17	1.0–6.0	0.51	−0.20	0.086	**0.229**	**0.152**	**0.191**	
Boys	3.75	1.21	1.0–6.0	0.29	−0.58	0.066	**0.232**	**0.184**	**0.162**	
Girls	3.50	1.11	1.0–6.0	0.72	0.43	0.082	**0.191**	**0.097**	**0.186**	
t-test	3.00**									
6. PA during PE	3.98	1.05	1.0–5.0	−0.76	−0.25	**0.214**	**0.305**	**0.266**	**0.301**	**0.212**
Boys	4.08	1.04	1.0–5.0	−0.92	0.03	**0.191**	**0.265**	**0.249**	**0.249**	**0.185**
Girls	3.89	1.05	1.0–5.0	−0.63	−0.39	**0.218**	**0.325**	**0.267**	**0.334**	**0.224**
t-test	2.50*									

Correlations in bold are significant at the 0.01 level, two-tailed tests; **p < 0.01; *p < 0.05. Cronbach’s alpha is listed diagonally (italics).

PA, physical activity; PE, physical education; part., participation.

### Design effect

The students in this sample were organized in classes. Hence, it was important to investigate whether individual responses could be accounted for by class-level belongingness. The assumption of independence may be violated through a cluster effect related to class belongingness (Hedges and Hedberg, 2007). The design effect (DEFF) is an index that reveals the extent to which the participants are independent of each other. DEFF is based on intraclass correlations (ICC), which reveal the degree to which variance in a variable can be explained by differences between clusters (Campbell et al., 2004; Koch, 1982). ICC were calculated using ANOVA (within and between group variance). DEFF was computed using the following formula: DEFF = 1 + (*m* – 1) × *p*, where m represents the average group size, and p represents the ICC (Donner and Klar, 2010; Snijders and Bosker, 2011). A cutoff value of two has been applied for the significance of DEFFs (Hox, 2002). The results of this analysis (Table 2) showed that only the DEFF for weekly PE participation. (DEFF = 3.08) was beyond the cutoff limit of two. However, this result probably occurred because different schools differ in terms of the number of obligatory and elective PE classes they provide for their students. As the remaining ICC/DEFF values were not significant, multilevel analysis was not conducted.

**Table 2:** Intraclass correlations (ICC) and design effects (DEFFs).

Variables	ICC	DEFF
PE teacher autonomy support	0.037	1.56
BPN-PE—Competence	0.008	1.13
BPN-PE—Autonomy	0.008	1.13
BPN-PE—Relatedness	0.010	1.15
Weekly PE participation	0.130	3.08
Physical activity during PE	0.015	1.24

### Structural equation model

**Figure 1:**
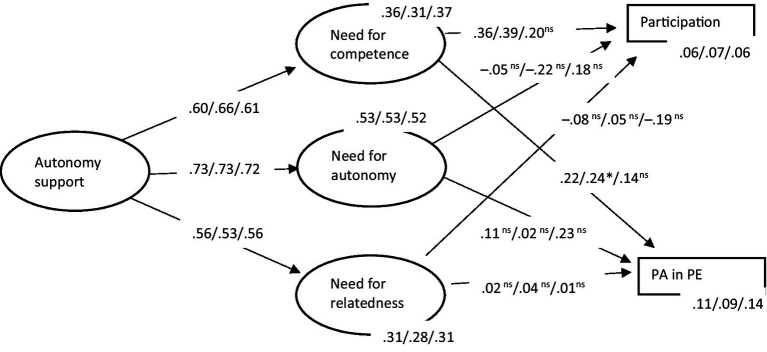
Structural equation model of autonomy support, basic psychological needs satisfaction, PE participation, and PA in PE with parameter values for total sample, boys, and girls. All paths significant *p* ≤ 0.01 except *p ≤ 0.05 and ns = not significant. Total effects (*r*^2^) are displayed. Covariances between variables were omitted for presentation clarity.

A structural equation model (Figure 1) was created using the abovementioned variables. This model assumed autonomy support to predict satisfaction of autonomy, competence, and relatedness needs, which in turn predicted both weekly participation in PE and PA during PE. The model produced acceptable fit indices (*χ*^2^ = 883.15, df = 161, *p* < 0.000, *χ*^2^/df = 5.49, CFI = 0.94, RMSEA = 0.07). Autonomy support positively predicted the need for competence, autonomy, and relatedness. The need for competence positively predicted weekly PE participation and PA during PE.

A multigroup analysis was conducted for a parameter comparison between boys and girls (Figure 1). The analysis showed acceptable fit indices for boys (*χ*^2^ = 482.46, df = 161, *χ*^2^/df = 3.41, CFI = 0.95, RMSEA = 0.07) and girls (*χ*^2^ = 650.84, df = 161, *χ*^2^/df = 4.04, CFI = 0.93, RMSEA = 0.08). A parameter value difference test (*z*-score) between boys and girls was performed. According to the guidelines for cutoff values (Diseth and Samdal, 2014), a *z*-score above 1.960 indicates significance at the 5% level, above 2.326 indicates significance at the 2% level, and above 2.576 indicates significance at the 1% level. The results showed that there were no significant parameter value differences between boys and girls, except for the parameter between need for autonomy and weekly PE participation, which had a higher value among boys than girls (*z* = 2.340, *p* < 0.05). However, the parameter values between these variables (autonomy and PE participation) were not significant for either of the subsamples.

## Discussion

The purpose of the present study was to examine how Norwegian 10th-grade students’ perceived autonomy support was associated with their satisfaction of the need for autonomy, competence and relatedness in PE, and how their BPN satisfaction was related to weekly PE participation and physical activity during PE, with gender-specific analysis. As a part of this study, we validated a Norwegian version of the Basic Psychological Needs in PE (BPN-PE) scale. In the current study, the SEM analysis demonstrated that autonomy support from PE teachers was positively associated with the satisfaction of the three BPN in PE for both genders. For boys, satisfaction of the need for competence was positively associated with weekly PE participation and physical activity during PE. Thus, these findings partly support Hypothesis 1. Furthermore, we found that there are gender differences, with boys scoring higher than girls on students perceived autonomy support and in their basic needs’ satisfaction (Hypothesis 2). Boys also scored higher than girls on weekly PE participation and physical activity during PE. In addition, we found the BPN-PE scale to be a valid and reliable instrument across genders (Hypothesis 3).

An essential step in the validation process of the BPN-PE scale is to continue testing its psychometric properties across countries and genders (Vlachopoulos et al., 2011; Badenes-Ribera et al., 2020). The CFA supported the hypothesized three-factor structure for the Norwegian version of the BPN-PE scale, in alignment with the theoretical assumptions in SDT stating that humans perceive competence, autonomy, and relatedness as three distinct psychological nutrients (Deci and Ryan, 2000). The factor structure is in accordance with previous validations from other countries (Cagas and Chasandra, 2014; Sánchez-Oliva et al., 2018; Vlachopoulos et al., 2011). We also found acceptable support for configural and metric invariance between genders, demonstrating that the underlying measurement structure of the BPN-PE scale is comparable for females and males. This was also demonstrated among Spanish (Sánchez-Oliva et al., 2018) and Greek (Vlachopoulos et al., 2011) students. Furthermore, the CFA of the autonomy support scale produced acceptable fit indices, and we also found satisfactory support for both configural and metric invariance across genders for autonomy support. Hence, we can make a meaningful comparison across genders for autonomy support. The positive association between autonomy support and BPN is also an indication of nomological validity of the scale (Cagas and Chasandra, 2014). In addition, the correlations between BPN and weekly PE participation and PA during PE support the predictive validity of the Norwegian BPN-PE scale. This adds support to the BPN-PE scale as a psychometrically sound measure of basic needs satisfaction in PE in Norway for both boys and girls.

In the SEM analyses students perceived autonomy support from teachers was moderately to strongly related to satisfaction of autonomy, competence, and relatedness, supporting the theoretical assumption that each of the three psychological needs is facilitated by autonomy support (Ryan and Deci, 2017). This finding adds to numerous studies across various contexts within the PE domain (Haerens et al., 2015; Pérez-González et al., 2019; Standage et al., 2003; Taylor and Lonsdale, 2010; Ulstad et al., 2016; Vlachopoulos, 2012) and highlights the role of PE teacher in creating a supportive environment, characterized by using noncontrolling language, try to understand how students cope with tasks, and provide supplementary help to foster the satisfaction of students’ basic needs in PE. A more in-depth understanding of this tendency is important as autonomy support from the PE teacher in addition to predicting higher BNS seems to nurture higher levels of motivation for PE, increase classroom engagement, and intention to be active (Pérez-González et al., 2019; Vasconcellos et al., 2020).

PE-based research has so far only to some extent focused on the direct effects of BPN on important student outcomes (Taylor et al., 2010), and only a few studies have assessed the relationship between the satisfaction of the BPN in PE and students’ involvement in PE. All BPN variables were significantly related to weekly PE participation and physical activity during PE in the bivariate correlation analysis for both genders, whereas in the SEM analysis, only satisfaction of the need for competence for boys predicted these PE outcomes, with weaker and non-significant associations for girls. This discrepancy between findings in the correlation analysis and findings in the SEM analysis is likely due to correlated predictors of weekly PE participation and PA during PE.

In line with our finding, previous research has also shown that the need for competence is the most important psychological need in terms of positive outcomes in PE, such as enjoyment, performance, and knowledge about the importance of PE (Gråstén and Watt, 2017), effort (Taylor et al., 2010), and intrinsic motivation (Ntoumanis, 2001; Standage et al., 2005). Vasconcellos et al. (2020) also found students’ satisfaction of the need for competence in PE to be most strongly related to students’ self-determined motivation for PE, indicating that a sense of self-efficacy in PE is especially related to wanting to be more involved in PE. Deci and Ryan (2000) hypothesized that the role of each psychological need may vary depending on the functional significance of the context. All three needs are important, but in different settings any of the needs will emerge to “take the lead” in terms of associations with a specific outcome (Ryan and Deci, 2017).

Taylor et al. (2010) found that all three psychological needs were positively associated with effort in PE among students in the United Kingdom. However, in agreement with our finding they found the need for competence to be the strongest and most consistent predictor of effort in PE, exercise intentions, and leisure-time physical activity.

One explanation for competence appearing to be the strongest predictor of the basic psychological needs in PE might be the uniqueness of school PE, as an arena of learning and demonstrating skills, that will amplify the need for competence as more important than the other two needs. If students feel that they perform well and succeed in tasks presented to them during PE lessons, they will feel competent (Ames, 1992; Standage et al., 2005). A recent review of qualitative studies supports this assumption by indicating that students experiencing poor competence in PE tend to reduce their involvement and participation in PE (White et al., 2021).

Regarding gender differences, the results showed that boys had higher mean level values than girls on all variables included in the study. The finding of higher levels of perceived teacher autonomy support among boys aligns with most studies from the PE context (Bagøien et al., 2010; Hosseini et al., 2020; Mouratidis et al., 2015). In line with previous research findings boys also scored higher than girls on the satisfaction of autonomy (Mouratidis et al., 2015) competence (Gholidahaneh et al., 2020; Mouratidis et al., 2015), and relatedness (Gholidahaneh et al., 2020; Haugen et al., 2021). Regarding relatedness satisfaction, our study found higher levels among boys compared to girls. This finding contrasts with existing literature, which has shown mixed results. Some studies report no gender differences (Mouratidis et al., 2015; Xiang et al., 2017) while others indicate that girls in upper secondary school (Bagøien et al., 2010) and in optional PE courses (Ntoumanis, 2005) score higher than boys on the need for relatedness. Thus, there could be gender differences related to age or the PE context. The abovementioned context specific aspects of Norwegian education may also have influenced the gender differences observed in students’ participation in PE. Our findings show that boys participate more frequently in PE than girls, consistent with previous research both in international (Martins et al., 2020) and Norwegian contexts (Haug et al., 2023; Lagestad et al., 2015; Lagestad et al., 2017). In the Norwegian context, girls have been found to skip PE classes without a valid reason more frequently than boys (Moen et al., 2018). A possible reason is that the Norwegian PE subject is dominated by competitive sports similar to leisure-time activities (Solenes, 2010; Säfvenbom, 2010) that are typically more attractive for boys than girls (Heradstveit et al., 2020). Accordingly, studies have shown that students are more likely to perceive higher BNS in PE if they attend leisure-time sports (Erdvik et al., 2020). A possible reason is that the Norwegian PE subject is dominated by competitive sports similar to leisure-time activities (Solenes, 2010; Säfvenbom, 2010) that are typically more attractive for boys than girls (Heradstveit et al., 2020). Accordingly, studies have shown that students are more likely to perceive higher BNS in PE if they attend leisure-time sports (Erdvik et al., 2020). The fact that sports and physical exercise activities dominate the teaching content in Norwegian PE (Moen et al., 2018; Solenes, 2010) could potentially also impact peer dynamics and, subsequently the satisfaction of BPN. Boys may form stronger social bonds through competitive sports, which again can enhance their relatedness satisfaction. Furthermore, it has been concluded that what are traditionally viewed as masculine values continue to be highly regarded in PE (Aasland et al., 2020). As in many other countries, traditional gender roles may influence students’ experiences in PE in Norway, and therefore impact how students perform and participate in PE. This may be attributed to various factors, including societal expectations, physical differences, and the activities provided in PE classes. If teachers unconsciously expect boys to perform better in PE, they might provide more autonomy support to boys, which can affect the experiences of both genders (Lagestad, 2017). One can speculate that this may potentially diminish girls’ relatedness satisfaction, as they may feel less supported and connected in the learning environment.

In addition, girls may experience more discomfort about sharing showers and changing rooms, inappropriate PE kit, or compulsory activities (Mitchell et al., 2015). PE in Norway is provided as a coeducational subject, with boys and girls attending the same class. Girls have been found to place a higher value on their physical appearance in such settings (O'donovan, and Kirk, 2008) and sometimes skip PE because they struggle to be physically active in front of others (White et al., 2021), particularly in front of boys (Røset et al., 2020).

## Limitations and conclusion

There are several limitations in this study that should be acknowledged. First, the design is cross-sectional and does not allow for examining causal relationships between the variables. Causality would require a longitudinal design, preferably with experimental control. Secondly, the data on physical activity were self-reported, which is known to have reporting bias (Nigg et al., 2020). However, validation studies on a similar item assessing overall PA indicate that self-reported PA levels correlate moderately with objective measures such as accelerometer data (Prochaska et al., 2001; Ridgers et al., 2012). Still, more objective measurements (e.g., accelerometers) are needed to more accurately assess the associations between BPN and students’ involvement in physical education classes. Time spent in PA during a PE lesson, will also depend on which activities the PE teacher provides and the intensity profile of the PE lesson (Zhou and Wang, 2019). We did not have information to control for this.

Despite these limitations, the present study adds knowledge to our understanding of students’ participation in PE. This understanding is important because physical activity during PE lessons tends to decline with increasing age (Hollis et al., 2017) and some students drop out of PE (Sälzer and Heine, 2016). Future research may utilize longitudinal or experimental designs to examine the causal relationships between perceived autonomy support from PE teachers, BPN satisfaction and behavioral outcomes in PE. It is also noteworthy that research has identified specific behaviors linked to the support of psychological needs (Ahmadi et al., 2023). Future research would benefit from measuring these behaviors in relation to needs support in physical education.

## Data Availability

The data analyzed in this study is subject to the following licenses/restrictions: the datasets presented in this article are not readily available. The University of Bergen is the data-bank manager for the international HBSC study. The data from the 2013/2014 survey is open access and available upon request. Requests to access these datasets should be directed to https://www.uib.no/en/hbscdata/113290/open-access.
